# Plantar Ulcerative Lichen Planus as a Therapeutic Challenge: A Review of Literature

**DOI:** 10.7759/cureus.87444

**Published:** 2025-07-07

**Authors:** Maria AlSulami, Khalid Alshareef, Abdulelah A Aldossari, Sultan N Alnasser, Asem Shadid, Lamia AlAkrash

**Affiliations:** 1 College of Medicine, Umm Al-Qura University, Makkah, SAU; 2 College of Medicine, King Saud bin Abdulaziz University for Health Sciences, Jeddah, SAU; 3 Department of Dermatology, King Fahad Specialist Hospital, Buraydah, SAU; 4 Department of Dermatology, King Khalid University Hospital, Riyadh, SAU; 5 Department of Dermatology, King Fahad Medical City, Riyadh, SAU

**Keywords:** chronic ulcer, erosive lichen planus, lichen planus, review article, ulcerative lichen planus

## Abstract

Plantar ulcerative lichen planus (PULP) is a rare variant of lichen planus (LP), with fewer than 20 cases reported in the literature. It presents as chronic, painful ulcers on the soles, often without other classic LP manifestations. The condition remains poorly understood, with unclear pathogenesis and no standardized treatment. Diagnosis relies on histopathological confirmation. This review aims to summarize the clinical features, comorbidities, diagnostic approaches, and therapeutic outcomes of PULP based on available literature.

A literature search was conducted on PubMed and Google Scholar up to December 2024. Relevant articles reporting cases of PULP with available clinical and treatment data were included.

A total of 18 cases were identified. The condition showed a strong female predominance, with a mean age of 63.7 years. Some patients had mucocutaneous involvement, while others presented with isolated plantar disease. Painful ulcerations affecting ambulation were the main clinical feature. Common comorbidities included autoimmune and metabolic disorders. Diagnosis was confirmed by histopathological features consistent with LP. Reported treatments included topical and systemic corticosteroids, calcineurin inhibitors, retinoids, cyclosporine, dapsone, phototherapy, biologics, surgical interventions, and Janus kinase (JAK) inhibitors such as tofacitinib, with variable outcomes.

PULP is a rare, chronic, and treatment-resistant condition that leads to significant morbidity. Histopathology remains essential for diagnosis, but therapeutic response is inconsistent and lacks standardization. Further studies are needed to better characterize the condition and establish evidence-based treatment guidelines.

## Introduction and background

Lichen planus (LP) is a chronic, immune-mediated inflammatory disorder that affects the skin, mucous membranes, hair, and nails [1]. It presents in various clinical subtypes including papular, hypertrophic, annular, vesiculobullous, and ulcerative depending on morphology and site of involvement [1]. The disease is primarily T-cell-mediated, targeting basal keratinocytes and resulting in characteristic violaceous papules or mucosal erosions. While most variants are self-limited, some, particularly ulcerative forms, follow a chronic, treatment-resistant course [2].

Erosive or ulcerative lichen planus (ULP) is an uncommon form of LP that mainly affects the oral cavity and genital regions [2]. ULP was initially identified and described by Friedman in 1921 [3]. Scarring alopecia of the scalp and involvement of mucous membranes are common [4]. Plantar involvement is very rarely observed in ULP, with only a limited number of cases documented in the literature [4]. Our review identified fewer than 20 reported cases of plantar ULP (PULP). ULP is marked by persistent, painful ulcers on the soles that can lead to significant disability [5]. These ulcers can occur in isolation without classic LP lesions in other areas of the body [4]. Unlike classic LP, which is believed to be mediated by the cellular immune response, the underlying mechanism of ULP remains unknown [6]. ULP is typically diagnosed based on histopathologic findings supporting the diagnosis [7]. The treatment of PULP remains a challenge due to its rarity and lack of a standardized treatment approach [5]. This review aims to provide an overview of PULP, including its clinical characteristics, diagnostic features, and treatment options reported in the literature. 

## Review

Methods

A literature search was conducted using PubMed and Google Scholar to identify published reports of PULP up to December 2024. The following terms were used: "ulcerative" OR "erosive" AND "lichen planus" AND "plantar" OR "foot" OR "feet" OR "sole". Filters were applied to include human studies and English language articles. Reference lists of relevant papers were also reviewed. Studies were included if they described ULP involving the plantar surface with sufficient clinical, histopathological, and therapeutic information. Articles without adequate data or describing non-plantar involvement were excluded. Two reviewers independently screened the titles and abstracts, followed by a full-text review. Data were manually extracted, including demographics, lesion characteristics, associated comorbidities, histopathologic findings, treatment modalities, and outcomes. Due to the nature of the included studies, no formal quality assessment was performed.

Results 

**Figure 1:**
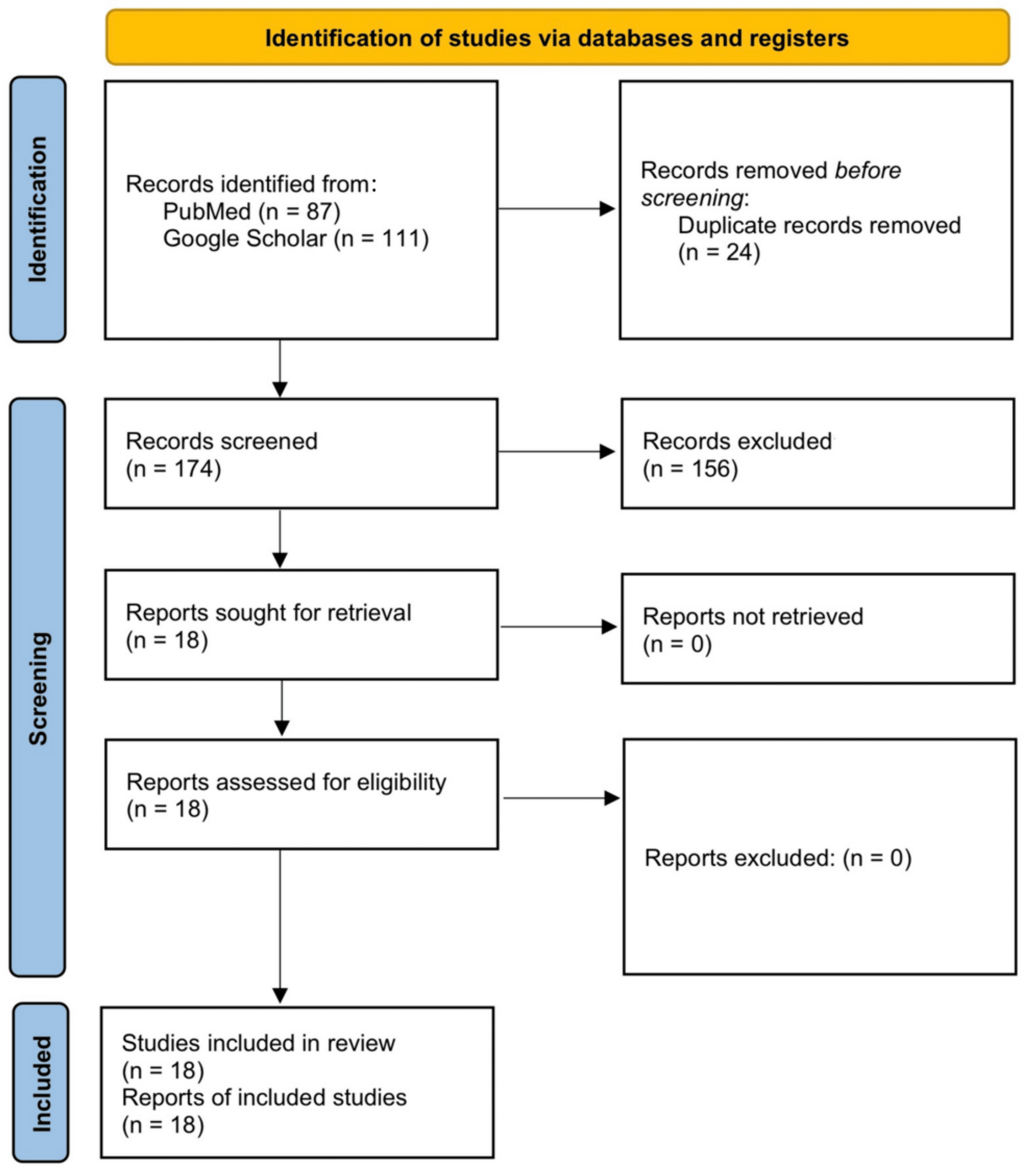
PRISMA 2020 flow diagram PRISMA: Preferred Reporting Items for Systematic Reviews and Meta-Analyses

After screening 174 records identified through our search, a total of 18 cases of PULP were included. The flow diagram of the screening process is shown in Figure 1. There was a marked female predominance (male-to-female ratio of 1:4.2), with a mean age of 63.7 years (range 38-81 years). Some cases presented with mucocutaneous LP, while others had isolated plantar involvement [8-11]. Associated comorbidities included autoimmune diseases such as Hashimoto's thyroiditis and Sjögren's syndrome, as well as metabolic conditions like type 2 diabetes mellitus, hypertension, dyslipidemia, and chronic kidney disease [4,7,12,13]. Hepatitis B serology was positive in only one case [5]. The disease exhibited a chronic and relapsing course, with an average duration of 8.2 years. Clinically, the most common presentation was painful, well-demarcated ulcerations on the soles and toes, frequently impairing ambulation [3-20]. The ulcers varied in appearance, ranging from irregular and punched-out lesions to erythematous, friable, or hyperkeratotic bases [5-7,12,16]. Some cases reported malodorous ulcers, scarring, syndactyly, and nail destruction [6-10]. A preceding history of pruritic keratoderma, violaceous plaques, or atrophic skin changes was noted in several patients, suggesting a potential progression from non-ulcerative to ulcerative disease [3,5,10]. Histopathological findings were consistent with LP, including hyperkeratosis, hypergranulosis, irregular acanthosis, basal layer vacuolar degeneration, and a band-like lymphocytic infiltrate at the dermoepidermal junction [3,4,8]. Additional findings such as apoptotic keratinocytes, melanin incontinence, Max-Joseph spaces, and saw-toothing of rete ridges were observed in some cases [3,6,13]. Treatments included topical corticosteroids, calcineurin inhibitors, and cyclosporine ointment, while systemic options ranged from immunosuppressants, retinoids, and biologics to phototherapy and skin grafting in severe cases. Responses varied, with no standardized approach. A summary of attempted treatments is provided in Table 1.

**Table 1:** Summary of administered and successful treatments for plantar ulcerative lichen planus (1985-present) PUVA: psoralen plus ultraviolet A light; UVA-1: ultraviolet A-1

Case no.	Author (year)	Administered treatments	Successful treatment (time to full/near-full epithelialization)
1	Mateu-Arrom et al. (2023) [11]	Topical steroids, topical calcineurin inhibitors, oral prednisone 45 mg/day for 6 weeks, methotrexate, cyclosporine, mycophenolate, tofacitinib 5 mg twice daily	Tofacitinib 5 mg twice weekly (3 weeks)
2	Bazargan et al. (2023) [9]	Cyclosporine tablets 100 mg three times a day, triamcinolone acetonide injection 5 mg/mL monthly, methotrexate 15 mg/week, tofacitinib 5 mg twice daily	Tofacitinib 5 mg twice daily (1 month)
3	Miotti et al. (2020) [7]	Topical steroid under occlusion, methotrexate 15 mg/week, autologous skin graft	Topical steroid under occlusion, methotrexate 15 mg/week, autologous skin graft (14 days)
4	Kandula et al. (2018) [6]	Local wound care, triple-therapy approach (oral prednisone 40 mg and clobetasol ointment 0.05% twice daily for two weeks, followed by oral doxycycline 100 mg twice daily with topical tacrolimus 0.1% twice daily)	Triple-therapy approach (4 months)
5	Kılıç et al. (2017) [4]	Topical treatment, phototherapy, cyclosporine A 3 mg/kg/day	Cyclosporine A 3 mg/kg/day (5 months)
6	Romero et al. (2016) [10]	Oral prednisone 1 mg/kg/day, clobetasol 0.05% with tacrolimus 0.1% twice daily, topical betamethasone and tetracycline, cyclosporine 50 mg three times a day, methotrexate 15 mg/week	No successful treatment
7	Sălăvăstru and Tiplica (2010) [12]	PUVA, systemic steroids, topical steroids, acitretin 0.5 mg/day, topical tacrolimus 0.1% twice daily	Topical tacrolimus 0.1% twice daily (4 weeks)
8	Goucha et al. (2011) [3]	Oral prednisone 1 mg/kg/day	Oral prednisone (3 weeks)
9	Al-Khenaizan and Al Mubarak (2008) [5]	Thalidomide, acitretin, cyclosporine, tacrolimus 0.1% ointment twice daily	Tacrolimus 0.1% ointment twice daily (4 weeks)
10	Mansura et al. (2006) [8]	Systemic and topical steroids, occlusion with Unna boot, antibiotics, acitretin, topical tacrolimus 0.1%, topical PUVA, medium-dose UVA-1 (60 J/cm²) five times per week	Medium-dose UVA-1 (60 J/cm²) five times per week (3 weeks)
11	Tsuboi and Katsuoka (2007) [13]	Topical zinc oxide ointment, steroid ointment, etretinate 30 mg/day	Etretinate 30 mg/day (2 months)
12	Meyer et al. (2005) [16]	Topical steroids, systemic retinoids, PUVA, methotrexate, topical tacrolimus 0.1% ointment twice daily	Topical tacrolimus 0.1% ointment twice daily (1 month)
13	Paçô and Silva (2001) [14]	Topical and systemic steroids, systemic retinoids, thalidomide, topical cyclosporine (50 mg/mL) in topical oily dressing	Topical cyclosporine 50 mg/mL in topical oily dressing (10 months)
14	Wollina et al. (2001) [15]	Topical corticosteroids, PUVA-bath, chloroquine, mycophenolate mofetil 2 g/day, topical betamethasone, UVA-1 (6 J/cm²), topical recombinant platelet-derived growth factor BB gel	Topical recombinant platelet-derived growth factor BB gel (5 months)
15	Patrone et al. (1998) [18]	Oral cyclosporine A, skin grafting	Skin grafting (immediate)
16	Dereure et al. (1996) [19]	Topical/oral steroids, photochemotherapy, oral etretinate, oral dapsone, oral cyclosporine A, oral thalidomide	Oral thalidomide (3 months)
17	Joshi et al. (1993) [17]	Etretinate 50 mg/day	Etretinate 50 mg/day (3 months)
18	Falk et al. (1985) [20]	Topical and systemic treatment, oral prednisone 40 mg/day, azathioprine 100 mg/day, hydroxychloroquine 200 mg twice daily, dapsone 50 mg/day	Dapsone 50 mg/day (9 months)

Discussion 

The predominance of PULP in older women suggests a potential hormonal or immunological role in disease pathogenesis. The association with autoimmune conditions and metabolic disorders further implies a systemic predisposition that may contribute to disease susceptibility. PULP follows a chronic and relapsing course as evident in many cases, with painful ulcerations significantly affecting mobility. The presence of preceding keratoderma or violaceous plaques in some cases suggests that PULP may evolve from non-ulcerative plantar LP over time, rather than being a distinct entity [3,9,10]. The coexistence of mucosal, palmar, or cutaneous LP-like lesions reinforces the idea that PULP belongs to a broader lichenoid disease spectrum [9,12,15]. Compared to classic LP, PULP demonstrates greater chronicity, higher resistance to treatment, and more severe morbidity, likely due to the mechanical stress on weight-bearing plantar surfaces. Histopathological findings confirm a lichenoid inflammatory process, with some cases displaying marked hyperkeratosis, ulceration, and dermal fibrosis, which may contribute to its persistent and refractory nature.

The heterogeneity in treatment responses reflects the absence of standardized therapeutic guidelines. Topical corticosteroids and calcineurin inhibitors are used in milder cases, but systemic treatments, including corticosteroids, immunosuppressants, and retinoids, are often required [7]. Cyclosporine A and methotrexate have shown partial or complete epithelialization over months [4,7]. Tofacitinib, a Janus kinase (JAK) inhibitor, has demonstrated rapid improvement in some refractory cases, offering a more targeted alternative [9,11]. Phototherapy (psoralen plus ultraviolet A light (PUVA), ultraviolet A-1 (UVA-1)) and skin grafting have been attempted with mixed results [8,18]. However, many cases remain resistant despite aggressive treatment [10]. Experimental therapies, such as recombinant platelet-derived growth factor BB gel, may offer new options [15]. Given the variability in response, individualized therapy is crucial.

A key limitation of this review is its reliance on case reports, which limits the ability to draw definitive conclusions about treatment efficacy and disease progression. The absence of large-scale studies or controlled trials makes it difficult to establish standardized treatment guidelines, and further research is needed to better define optimal therapeutic strategies. Future studies should focus on evaluating JAK inhibitors, biologics, and combination therapies to establish evidence-based guidelines.

## Conclusions

PULP is a rare, chronic, and disabling variant of LP that primarily affects older women and often follows a relapsing course. It presents with painful, treatment-resistant ulcerations that significantly affect mobility. Various treatments have been attempted, including topical corticosteroids, calcineurin inhibitors, cyclosporine ointment, systemic corticosteroids, methotrexate, retinoids, dapsone, cyclosporine A, thalidomide, phototherapy, skin grafting, and, more recently, JAK inhibitors such as tofacitinib. While some cases showed partial or complete response, many remained refractory, and no standard protocol exists. JAK inhibitors have shown encouraging results and may offer a promising therapeutic option. Larger case series and controlled studies are needed to guide evidence-based management and improve outcomes.
